# Supplementary figures and images for: Transcriptome analysis during 4-vinylcyclohexene diepoxide exposure-induced premature ovarian insufficiency in mice
Source: PeerJ. 2024 Apr 18;12:e17251. doi: 10.7717/peerj.17251 (PMC11032656; doi:10.7717/peerj.17251)

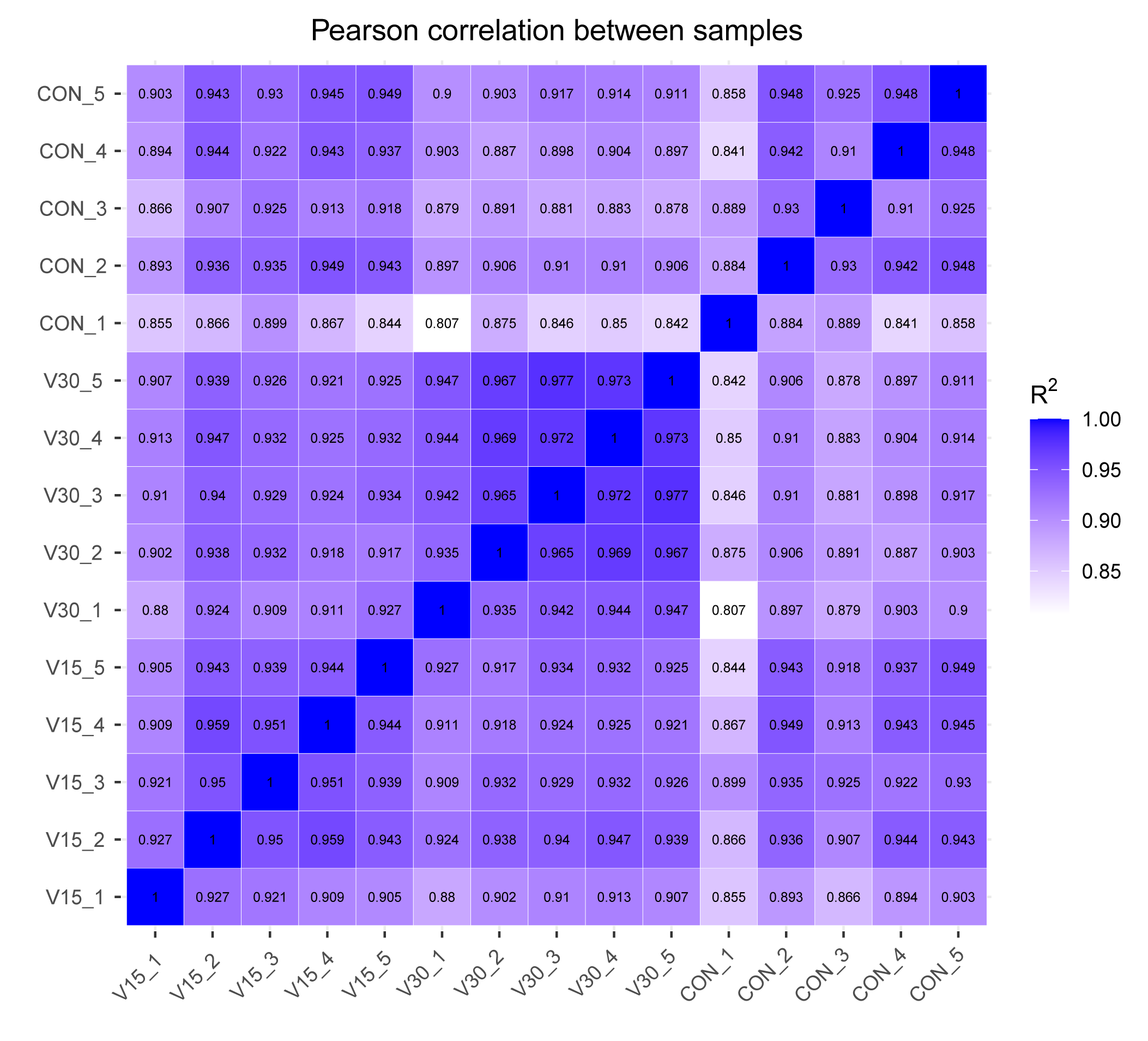

Supplement: Figure S1 — The correlation coefficients of the samples within and between groups were calculated based on the FPKM values of all genes in each sample and plotted as a heat map. The closer the correlation coefficient is to 1, the higher the similarity of expression patterns between samples. [file peerj-12-17251-s001.png]

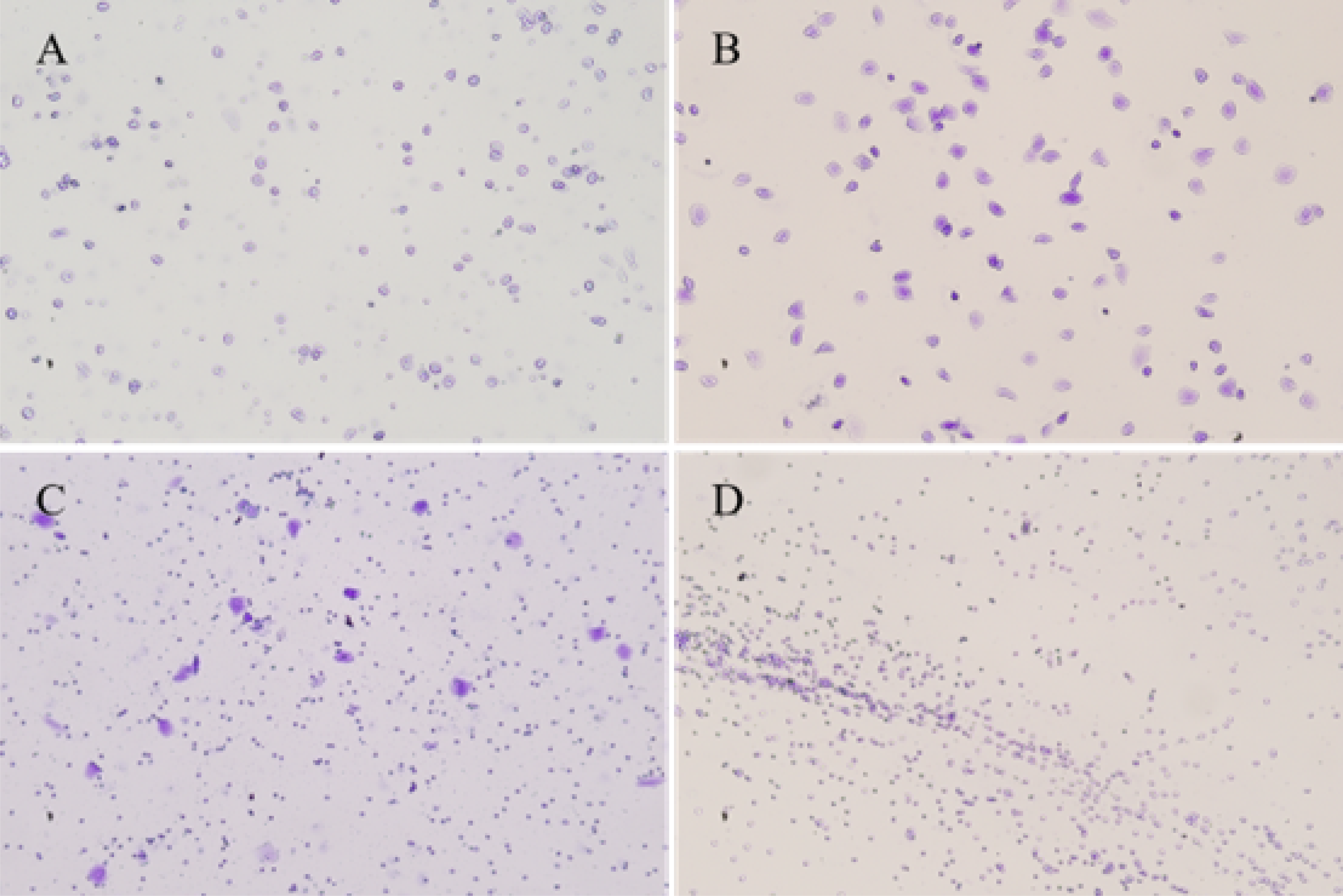

Supplement: Figure S2 — A. Proestrus; Characterized by a large number of nucleated epithelial cells, and a few keratinized cells are seen. B. Estrus; Characterized by large numbers of keratinized cells. C. Metestrus; Characterized by the presence of both keratinized cells and leukocytes. D. Diestrus; Characterized by the presence of large numbers of leucocyte and mucus. [file peerj-12-17251-s002.png]
