# Supplementary material for: Transcriptome analysis during 4-vinylcyclohexene diepoxide exposure-induced premature ovarian insufficiency in mice
Source: PeerJ. 2024 Apr 18;12:e17251. doi: 10.7717/peerj.17251 (PMC11032656; doi:10.7717/peerj.17251)
Supplement: Table S1 — *Compared to the control group, P < 0.05. [file peerj-12-17251-s003.docx]

Supplementary Table 1. Statistics on the estrous cycle of mice during modelling period

| Group | mice | oestrus cycle | | |
| --- | --- | --- | --- | --- |
|  |  | Number of regularities | Number of irregularities | Irregular rates |
| CON | 15 | 12 | 3 | 20.00% |
| VCD | 15 | 2 | 13 | 86.67%^*^ |

*Compared to the control group, P<0.05
